# Analyzing Awareness on Risk Factors, Barriers and Prevention of Cervical Cancer among Pairs of Nepali High School Students and Their Mothers

**DOI:** 10.3390/ijerph16224382

**Published:** 2019-11-09

**Authors:** Kritika Poudel, Naomi Sumi

**Affiliations:** 1 Graduate School of Health Sciences, Hokkaido University, Sapporo, Hokkaido 060-0812, Japan; kpoudel01@gmail.com; 2 Faculty of Nursing, Hokkaido University, Sapporo, Hokkaido 060-0812, Japan

**Keywords:** cervical cancer, awareness, students, mothers

## Abstract

Providing information on increased cancer risks associated with certain behaviors might encourage adolescents to initiate protective behaviors. This study firstly determined the knowledge of risk factors and prevention of cervical cancer. Secondly, it checked an association between mothers’ screening practice and student’s knowledge. A descriptive, cross sectional study was conducted among 253 pairs of high school students and their mothers. Knowledge on cervical cancer was significantly lower among students and mothers. While cancer screening tests, maintenance of hygiene were considered as major preventive measures for cervical cancer, human papilloma vaccine was the least considered preventive measure. Students who were female, attended discussions on cancer and had a healthy diet had better awareness of cancer. Mothers of female students had better knowledge about cervical cancer than mothers of male students. Less perceived susceptibility and lack of knowledge were major obstacles among mothers, limiting cervical cancer screening to 15%. Although association between knowledge of students and screening practice of mothers was not clear, it was observed that cancer communication increased awareness of cervical cancer in both groups. Our findings showed a strong need for school-based cancer education program to address the issues of human papillomavirus vaccinations, cervical cancer risk and screening.

## 1. Introduction

Cervical cancer is considered the fourth most common form of cancer among women, with an estimated 570,000 new cases in 2018, comprising 6.6% of all female cancers. Approximately 90% of deaths from cervical cancer occurred in low- and middle-income countries [1]. In Nepal, cancer of the cervix uteri is a major cause of death, resulting in 18.4% of all deaths [2]. It is the most frequent cancer among women between 15 and 44 years of age. Every year, 2332 Nepali women are diagnosed with cervical cancer and 1367 succumb to the disease [3,4]. The incidence of cervical cancer is 24.2 per 100,000, which makes Nepal one of the countries with the highest cervical cancer mortality rate in South Asia [4,5,6]. However, due to a lack of a national population-based cancer registry in Nepal, the numbers may be underreported.

The risk factors for cervical cancer are early incidence of first sexual intercourse, multiparity, tobacco smoking and multiple sex partners [7]. Cervical cancer is caused by sexually acquired infection by certain types of Human papillomavirus (HPV). The primary cause is the result of infection with HPV type 16 and 18. HPV 16 and 18 cause 70% of cervical cancers and pre-cancerous cervical lesions. With immunological treatment, most lesions disappear after 6 to 12 months. Although most HPV infections clear up on their own and most pre-cancerous lesions resolve spontaneously, all women are at risk of the HPV infection becoming chronic and the pre-cancerous lesions progress to invasive cervical cancer. It takes 15 to 20 years for cervical cancer to develop in women with normal immune systems. It can take only 5 to 10 years in women with weakened immune systems, such as those with untreated Human Immunodeficiency Virus (HIV) infection. Infection with certain HPV types also causes a certain proportion of cancers of the anus, vulva, vagina, penis and oropharynx, which are preventable using similar primary prevention strategies as those for cervical cancer [8]. Reproductive and sexual factors, such as multiple sexual partners, multiparity and early age at first intercourse and first child birth, are attributable to an increased risk of HPV infection [9,10]. Behavioral factors, such as smoking and obesity, are also related to increased risk of cervical neoplasia [10].

Several tests are being used in cervical cancer screening. The World Health Organization suggests the Papanicolaou (pap) test as an effective test to reduce the incidence and mortality of cervical cancer [8]. Although the pap test has been the primary screening method, its availability and accessibility are limited in Nepal, which lacks human and financial resources [5]. The HPV test provides highly sensitive results; however, its high cost has hampered its use in Nepal. Visual inspection with acetic acid (VIA) is used for potential cervical dysplasia in Nepal [11]. Lately, with VIA being convenient, precise and reasonable in price, the use of alternate screening methods has been encouraged [5]. Regardless of the test being used, the key to an effective program is to approach the vast population of women with quality screening and treatment [12].

Licensed medical doctors, gynecologists and trained registered nurses are responsible for conducting cervical cancer screening tests in different clinical settings. Women aged 30 to 60 years should take the screening test at least once a year, but these cancer screening tests are not free in Nepal. Cervical cancer is highly treatable if diagnosed in its early stages; however, in Nepal most of the women present in hospital once the cancer has become invasive, resulting in poor prognosis. The primary reasons of delay in treatment are lack of proper awareness and education on screening in communities, limited number of health workers, and lack of affordable preventive services [3]. Cultural norms, embarrassment, poor access to health posts, fear of cancer, lack of knowledge and the prohibition of gynecological tests among unmarried women were some of the individual barriers reported [11,12]. Furthermore, insufficient skilled health personnel, instruments and access to proper laboratories are other health care- system-related barriers to cervical cancer screening [11].

Cervical cancer also concerns adolescents, as Nepal has one of the highest rates of child marriage in Asia for both girls and boys. Although the legal age of union for both sexes is 20 years, more than a third of young women aged 20–24 report that they were married by the age of 18, and just over one in 10 report being married by the age of 15. Moreover, just over one eight of Nepali women have babies before the age of 18, putting girls at a higher risk of death or injury during childbirth, with concomitant reproductive health threats [13].

There is little understanding about the knowledge levels of the adolescent population regarding cervical cancer in Nepal, even though they are the population most exposed to the related risk factors. Young people are at high risk in connection with their age of first intercourse, increase in unwanted pregnancies and abortions, multiple sexual partners and incidence of sexually transmitted diseases (STDs) [14]. These behaviors are predisposing factors for cervical cancer in the future. According to the Nepal census, 24.19% of the population are in the 10–19 years age group [15]. Adolescents are acquiring new habits and behaviors during their adolescence phase. It is important to educate adolescent boys and girls about cervical cancer prevention and screening methods. Providing adolescents with information about the increased cancer risks associated with certain behaviors may be one way to encourage protective behaviors that provide the foundation for a healthy adulthood [16]. Therefore, assessment of their awareness on the causes of cervical cancer, HPV infection and vaccination can lead to successful reductions in the increasing burden of cervical cancer [17].

In South Asian countries, mothers play an important role in the family. Their education, awareness, health beliefs and practices influence the health awareness and practices of their children. A Japanese study suggested that acceptance of the HPV vaccine was mainly determined by mother’s perception of risks and benefits rather than the daughter’s wishes [18]. Regarding pap testing behavior, mothers may be an important socializing agent for their daughters [19]. To check the association of mothers’ screening practice and its effect on knowledge of adolescents, mother and student were examined in pairs in this study.

One of the popular models in health education is the Health Belief Model (HBM). The HBM is a conceptual framework to understand health behavior and the possible reasons for non-compliance with recommended health actions [20]. This model has the major subscales of perceived susceptibility, severity, benefits and barriers. The likelihood that an individual will act to prevent or detect disease is determined by several factors: perceived susceptibility to the health condition, perceived severity of the health threat, perceived benefits of performing the health behavior and perceived barriers to performing this behavior [21]. We used the barriers subscale in our study to understand the inhibitory factors of mothers related to screening.

This study firstly determined the knowledge of risk factors, symptoms and the prevention of cervical cancer. Secondly, it aimed to check an association between mothers’ screening practice and high school student’s knowledge. We hypothesized that knowledge, cervical cancer screening practices and beliefs of mothers would be associated with cervical cancer awareness of their children.

## 2. Materials and Methods

### 2.1. Data Collection

A descriptive cross-sectional study was conducted among three secondary schools of the Lalitpur metropolitan area of Nepal. A convenience sampling method was used. Only government high schools in the Lalitpur metropolitan were chosen for the study, and permission letters were sent to all school principals. Only those schools that permitted our research were included in this study. The primary participants of this study were adolescents, as students acquire new habits at this stage. Healthy and unhealthy habits learned during adolescence help shape their future lifestyles. Furthermore, students are likely to drop out after grade 10 because of factors such as work, family responsibilities and marriage. Hence, grades 9 and 10 were our primary target in this study.

An envelope containing the permission letter, consent form, questionnaire and a pen was prepared for mothers. To determine the mother-student pairs, a code was written on the envelope that was only known to the researchers. The permission letter was sent to mothers as well as students. The permission letters were sent to 335 mothers; 258 returned the questionnaire and 270 returned the permission letter along with the questionnaire. Mothers who could read and write completed the questionnaires alone. Mothers who could not read and write were requested to have the questionnaire completed by their husbands or a reliable family member, excluding children or minors. Based on the code provided, the questionnaire was distributed to the 270 students whose mothers approved their participation in this research. After excluding missing data, 253 pairs of students and mothers were included in this study. The data were collected from October to December, 2017.

### 2.2. Questionnaire

In order to understand the awareness on cervical cancer, the questionnaire was prepared based on the review of several studies. The questionnaire consisted of items on socio-demographic data, personal health information and knowledge of risk factors, symptoms and preventive measures. The questionnaire provided to the mothers included additional questions related to barriers and screening practices. The knowledge of risk factors and preventive measures were combined for a knowledge score ranging from 0 to 21. A correct answer was scored 1, while 0 was given for incorrect answers. A question related to risk factors was prepared using the Cervical Cancer Knowledge Prevention 64 questionnaire [22]. Another question focusing on the perceived barriers was developed based on the Health Belief Model (HBM). Questionnaires were translated into Nepali and then reverse-translated into English by experts. Content validity was established by an extensive literature review, and consultations with a research advisor and medical doctors. The tool was reverse translated into English by two experts to ensure retention of the same concepts. Reliability was measured in a pre-test. The pre-test was conducted with 20 students and mothers in similar settings to check the validity of the questionnaire. Some questions were edited and simplified based on the feedback received. After the pre-test, the internal consistency of the questionnaires was estimated with Cronbach’s alpha, whose value was 0.81. The reliability was re-evaluated in the whole analyzed sample. The value of Cronbach’s alpha was 0.75. As the estimate was greater than 0.70, it was considered acceptable for the study.

### 2.3. Statistical Analyses

A *t*-test and Analysis of variance (ANOVA) were used to examine the relationship between knowledge scores and the independent variables. Multiple linear regression analyses were performed on variables that were statistically significant during *t*-test and ANOVA to find the predictor of awareness among students and mothers. The level of significance was set at 0.05. All analyses were performed using the IBM SPSS Statistics for Windows software version 22.0 (IBM Corp., Armonk, NY, USA).

### 2.4. Ethical Considerations

This study was approved by the ethical review committee of Hokkaido University, Japan and the Nepal Health Research Council (134/2019). Before conducting research, written consent was obtained from all participants. The names, addresses, and contact numbers of students, and names of schools and parents who would disclose their personal information were not collected during data collection. Each student was given a unique code number by a researcher to pair the students with their mothers. The participants were free to quit at any time during data collection. Data privacy was maintained after data collection. The data will be stored for five years.

## 3. Results

A total of 253 pairs of mothers and students were included in this study. The mean ages of the students and mothers were 15.0 ± 1.0 and 40.4 ± 5.5 years, respectively.

### 3.1. Demographic Information

#### 3.1.1. Students

Out of 253 students, 50.6% of the students were female and 54.2% were studying in grade 9; 38.7% of the students had the habit of doing some sort of physical exercise; around 41.9% of the students had the habit of eating healthy foods (fruits and vegetables, lentils, high-fiber food) at least 3–4 times a week; about 22.1% of the students reported a family history of non-communicable diseases (NCDs); about 26.1% of the students said they had talked about cervical cancer with others, such as friends, family or both. The most common source of information about cervical cancer was television (56.7%), followed by the Internet (47.4%) and friends (26%). The mean knowledge score of students was 11.11 ± 4.64.

#### 3.1.2. Mothers

The mean age of mothers when they married was 19.7 ± 3.3 years. The median number of children that mothers had was 2. A total of 41.1% mothers were illiterate. About 62.8% mothers were less than 40 years in age. Around 10.7% and 23.3% of the mothers had the habit of smoking cigarettes and drinking alcohol, respectively. About 60.1% of the mothers had not talked about or discussed cervical cancer with anyone, while 22.5% had talked about it with their family, 9.5% with friends, and 7.9% with both family and friends. The most common source of information about cervical cancer among mothers was television (60.5%). Only 32.4% of mothers had heard about cervical cancer screening and 15% had taken the screening test. The mean knowledge score for mothers was 10.34 ± 4.77.

### 3.2. Knowledge on Risk Factors of Cervical Cancer

**Figure 1:**
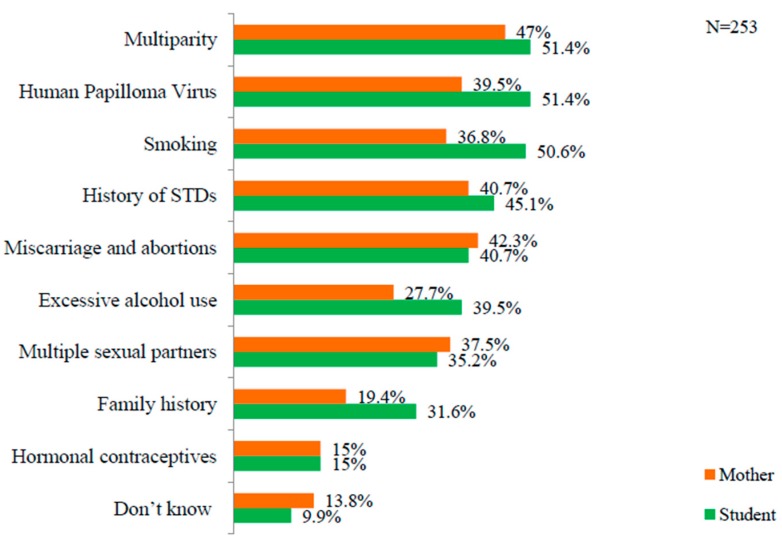
Knowledge of mothers and students about the risk factors of cervical cancer.

Multiparity was considered the main risk factor for cervical cancer by both students (51.4%) and mothers (47%). Use of hormonal contraceptives was considered the least important risk factors by both students and mothers. Detailed information is provided in Figure 1.

### 3.3. Knowledge on Prevention of Cervical Cancer

**Figure 2:**
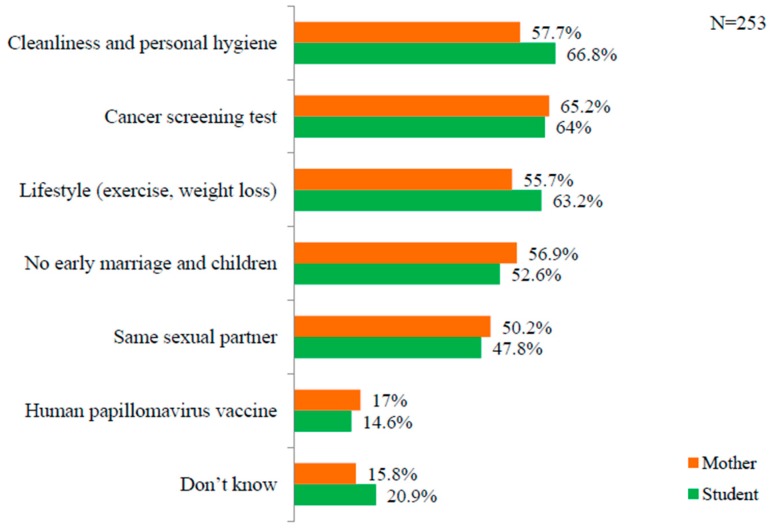
Knowledge of mothers and students about the prevention of cervical cancer.

The students considered cleanliness and personal hygiene (66.8%) the major preventive measures. For mothers, cancer screening tests (65.2%) were indicated to be the most effective preventive measure. It was interesting to see that the HPV vaccine was the preventive measure least considered by both students (14.6%) and mothers (17%). Further information is provided in Figure 2.

### 3.4. Total Cervical Cancer Knowledge Score

#### 3.4.1. Students

Female students had better knowledge than males (*p* < 0.01). Education level played an important role in knowledge. Grade 10 students had better knowledge than grade 9 students (*p* < 0.02). Students who followed a healthy diet (*p* < 0.00) were more knowledgeable.

Similarly, students, who had a habit of talking about cancer with family or friends, had more knowledge about cervical cancer than students who did not talk about cancer with anyone (*p* < 0.00). 

Family history of chronic illness, cervical cancer screening habit of mothers and area of residence showed no association with knowledge among students. Further information is provided in Table 1.

Multiple linear regression (*R*^2^ = 0.10, *p* = 0.00) that was performed to identify the predictors of awareness among students showed that cancer talk (*β* = 0.19, *p* = 0.00) was a major predictor. Beside cancer talk, education level (*β* = 0.19, *p* = 0.01), sex (*β* = 0.16, *p* = 0.01) and healthy diet consumption (*β* = 0.13, *p* = 0.04) were other important predictors of awareness. Further information is provided in Table 2.

#### 3.4.2. Mothers

Mothers who exercised were more knowledgeable than mothers who did not exercise at all (*p* < 0.00). Similarly, education level, age at the time of marriage and living area had significant effects on awareness (*p* < 0.00). 

Mothers who had taken a cervical cancer screening test (mostly a pap test) had better knowledge levels than mothers who had not taken any screening test yet (*p* < 0.00). Mothers who had shared information or talked about cervical cancer with family members or friends had better knowledge than mothers who had never talked about cervical cancer with anyone (*p* < 0.02). Mothers of female students had better knowledge about cervical cancer than mothers of male students (*p* < 0.05). Further information is provided in Table 3.

Multiple linear regression was performed on variables (education level, area of residence, exercise habit, cancer screening habit, talk on cancer, age during marriage and sex of children) to find the predictor of awareness among mothers. Multiple linear regression (*R*^2^ = 0.17, *p* = 0.00) showed that cancer screening habit (*β* = 0.17, *p* = 0.01) as the strongest predictor. Exercise habit (*β* = 0.16, *p* = 0.01) was the second predictor of awareness followed by education level (*β* = 0.15, *p* = 0.03) and area of residence (*β* = 0.13, *p* = 0.04). Further information is provided in Table 4.

Mothers were also asked questions concerning barriers. Mothers with a low knowledge level showed less perceived susceptibility towards cervical cancer (*p* < 0.01). Lack of information about cervical cancer was another barrier among mothers (*p* < 0.00). Further information on barriers to screening is provided in Table 5.

## 4. Discussion

This study was conducted among high school students and their mothers. Although there are several studies done in Nepal to explore understanding of women, little research has been done to explore adolescent boys’ and girls’ understanding regarding cervical cancer. Hence, this study assessed cervical cancer awareness among both sexes in Nepal. Furthermore, this is the first study conducted in Nepal, pairing mothers with their children, adding information about cancer communication in families and linking student knowledge and mothers’ screening practices.

### 4.1. Student’s Knowledge and Opinion

Multiparity (51.4%) and smoking (50.6%) were considered major risk factors by students. This finding is similar to those of a study conducted in Ethiopia, where having multiple sexual partners, early age at sexual intercourse and cigarette smoking were regarded as major risks in contracting cervical cancer [23]. It was interesting to find that only 35.2% of the students considered having multiple sexual partners as a risk factor for cervical cancer. This is an alarming sign in that most students do not know about the risks of having multiple sexual partners. Prevalence of premarital sexual intercourse and risky sexual behavior is an emerging issue among Nepali students [24]. School-based sexuality education might help many youths, as their sexual partners are also students.

The total knowledge score of students was low in this study. This finding was similar to that of a Japanese survey, in which more than 50% of students were not familiar with cancer topics [25]. This suggests the need to expand educational curricula related to cancer.

Only 14.6% of the students and 17% of the mothers in this study were aware of the availability of HPV vaccines. It was interesting to note that most mothers and students did not consider HPV vaccination as being able to prevent cervical cancer. This might be because of lack of awareness of the latest technology developed to screen for cervical cancer and to prevent it. The vaccine for cervical cancer is effective if received before the onset of sexual activities at 9–13 years [7]. In Nepal, several campaigns and tests have been conducted involving vials donated intermittently without cost by different national and international organizations. The issues associated with the HPV vaccine in Nepal might be affordability, maintenance of a cold chain and public awareness [26]. There is a strong need for education intervention among boys, girls and their parents to change attitudes toward HPV vaccination [16,27]. The HPV vaccination program is in its pilot phase in Nepal. The start of routine HPV vaccination immunization programs is yet to be decided by the Government of Nepal (GoN); however, GoN plans to provide free health screening services and full detailed coverage of a vaccination campaign against cervical cancer in fiscal year 2019–2020 [28].

Students who had high knowledge levels were from grade 10, which might be because students in this grade have read about cancer recently in their classes. Cancer education is included in the grade 10 curriculum. Students who had high knowledge levels had the habit of eating a healthy diet at least 3–4 times a week. Exercise and healthy diet are important in maintaining a healthy life and preventing diseases. Increasing motivation is the first and most important step among non-motivated people who are yet to engage in a healthy behavior [29].

It was seen that female students were more knowledgeable than male students. This might be because of the interests, motivations and curiosity about diseases that are common among women. Even though knowledge about cervical cancer was low among boys, 76% of the boys in this study strongly felt the need for cervical cancer education.

This finding is similar to a study conducted among college students in India that showed that girls had greater knowledge about cervical cancer than boys [30]. However, a Kenyan study showed that male students were interested in learning more about cervical cancer and its association with them [31]. The interest of boys plays an important role in encouraging female family members, including wives and mothers, to receive cervical cancer screening. Several programs integrating both male and female students could be helpful in addressing not only cervical cancer prevention but also reproductive health.

Students with a high knowledge level had the habit of talking to others about cancer. This might be because their knowledge was high enough that they could talk to others. This showed that cancer education among students will increase talking with others. Our study showed cancer talk as the strong predictor of knowledge among students. A Japanese study revealed that peer education helped participants obtain greater knowledge about cervical cancer. Friends are the most influential person during adolescence, and peer education might help in improving screening rates among young women [12]. During adolescence, peers play a crucial role in changing the personality, attitudes and behavior of a person [24]. Hence, cervical cancer education programs including peer group activities might be more effective in the adolescent group.

### 4.2. Mothers’ Knowledge and Opinions

The mean risk score of mothers was low, with a mean of 10.34 ± 4.77 (range: 0–21). Lower knowledge might be related to a lack of proper information on cervical cancer, less access to health- related programs and events, unawareness of cultural barriers, etc. Similar findings were found in a study conducted in China, where a lower level of knowledge was associated with insufficient health education, limited access to mass media and less communication between women and health personnel [32].

Mothers who had had cancer communication with friends, family or both had greater awareness of the risks and prevention of cervical cancer. This revealed that women who had discussed cervical cancer with other people were more active in taking screening tests. They also had greater motivation and reduced barriers and encouraged other women to take screening tests. A study conducted among Australian women in Victoria to understand the awareness of HPV showed that only 14.8% of women had ever discussed HPV with a friend [30]. By engaging family members in the awareness campaign, an education program promotes health awareness as well as helps in developing healthy habits, eventually leading to improving quality of life.

The higher the education level, the better was the knowledge among mothers. This showed that education is necessary to create awareness of diseases or any other infirmities. These mothers also had a greater practice of screening than mothers with low education levels, which might be due to higher-educated mothers being more conscious about their health and more aware about cervical cancer. Several studies have supported the positive relationship between education level and high knowledge in Nepali context [33,34].

Mothers who exercised at least three–four times a week had greater knowledge about cervical cancer. Their major concern was the associated risk of obesity with cancer. Although more than 79% mothers did not have a habit of regular exercise, they considered performing daily household chores as their main exercise.

This study showed that mothers residing in urban areas had better knowledge about cervical cancer. The reason might be that urban areas have more facilities for health care and more campaigns and promotions on cancer screening than rural areas. This finding was similar to another study in Nepal that found that women in urban areas had better knowledge, practices and attitudes [35]. In 2003, around 2.8% of women aged 25 to 64 years were screened and majority of them were living in urban areas [4].

Mothers who married after reaching 20 years of age had somewhat better knowledge of cervical cancer than mothers who married before reaching the age of 20. This might be because they were more aware of their reproductive health and had had more exposure to cervical cancer information.

Mothers who had done cervical cancer screening tests had better knowledge about it than mothers who had not taken a screening test yet. Additionally, our study showed cancer screening habit as a strong predictor of knowledge. While about 32.4% of the mothers had heard about screening, only 15% had taken the cervical cancer screening test. The findings are similar to those of another study conducted among Nepali women, where 18.3% of the women surveyed had participated in cervical cancer screening. [33]. Another study showed about 95% of Nepali women aged 21–65 years had never taken a cervical screening test [36]. In developing countries, only 5% of eligible women underwent cytology-based screening in a 5-year period [37]. This shows that most Nepali women are not aware of the advantages of cervical cancer screening. In addition, laboratory services as well as pathologists to evaluate cervical smears are practically non-existent in rural parts of Nepal, which means that women must travel to secondary or tertiary centers to receive appropriate testing, limiting the screening uptakes [36].

Comprehensive cervical cancer control includes primary prevention (vaccination against HPV), secondary prevention (screening and treatment of pre-cancerous lesions), tertiary prevention (diagnosis and treatment of invasive cervical cancer) and palliative care. Carefully designed messages are necessary in order to educate communities, parents, teachers, adolescents and other stakeholders about the HPV vaccine, HPV infection and cervical cancer and the availability of services. Programs can be quickly undermined by rumors and misinformation if the reasons for targeting girls only are not fully and sensitively communicated. Therefore, educating men, including fathers and boys, about HPV vaccines and cervical cancer is particularly important in this regard [38]. Knowledge is primarily gained through education programs, which is supposed to be a precursor for behavior. However, attitude also plays an important role in formulating behavior. Therefore, an awareness program should also uplift the screening attitude of women. Women health professionals and Female Community Health Volunteers (FCHVs) can play a crucial role in overcoming issues related to shyness and cultural issues, as Nepali women are hesitant to be examined by male health professionals [39]. There is strong urge to develop a woman-friendly screening program to increase the screening uptake.

Mothers of female students had better knowledge about cervical cancer than mothers of male students. This might be because female students were more comfortable in sharing information about cervical cancer with their mothers. Education programs collaborating with mothers and children might help in increasing their perception of susceptibility to cervical cancer and in increasing their cancer screening uptake in the future. Students can share knowledge about the education program with their mothers, increasing their mothers’ knowledge levels, resulting in the diffusion of cancer knowledge, which supports the Learning Partner Model. The Learning Partner Model is a framework that supports the diffusion of health information to enhance early cancer detection programs [40].

### 4.3. Clinical and Educative Implication

Cervical cancer is an important issue in Nepal; hence, proper strategies should be developed to increase women’s participation in screening programs [41]. Lack of proper information and less perceived seriousness were barriers hindering cervical cancer screening among mothers. Public education programs are effective in increasing screening uptake and maintaining screening behaviors [32]. These barriers should be addressed using qualitative as well as longitudinal studies to check the types and stability of barriers. A Jamaican study showed that various factors like socioeconomic status, low perceived risk of disease, fear of being diagnosed with cervical cancer and fear of pain and embarrassment affect the uptake of cancer screening tests [42]. Another Nepali study showed that females’ decision to receive screening were affected by the societal norms of a patriarchal society, the availability of health centers and a lack of support from family members. This might suggest that Nepali women have less power to take initiatives for their health [11]. Hence, this showed the need for developing programs that could address the health beliefs of all family members. Self-collected cervical sampling methods could be another option to increase screening participation among women, making screening convenient and private. This can address barriers related to blaming and shaming and the method of screening in areas with limited health services. However, a lack of proper and well-equipped laboratories and skilled personnel are issues related to self-sampling techniques. A Nepali study focused on the self-collected sampling methods among women of rural areas and strongly suggested the need for additional research in this area [6].

The health system in Nepal faces unsettling challenges such as non-uniformly distributed health care services, the shortage of essential medicines, poorly regulated private providers, a lack of actual budget for health and poor retention of human resources in rural areas. Addressing barriers to health services requires urgent interventions at the population level [43]. While the government has yet to address all these challenges, health care providers can play an important role in effective counseling and explanation to people of screening tests and vaccines. A sympathetic approach, good communication skills, respectful behaviors, performance of less painful screening tests and health care providers can help to improve health-seeking behaviors among Nepali women.

Many researchers have focused on the importance of increasing public awareness of the warning signs and symptoms of cancer, as well as preventive measures [41]. The Government of Nepal is actively organizing several health camps and screening campaigns in different parts of the country. However, the expected screening coverage is not satisfactory. The higher the screening barriers, the less likely women are to take the chance of screening [33]. As the perception of participants’ susceptibility to cervical cancer can affect screening, efforts to increase the screening rate should focus on informing women of their susceptibility, which will help to enable the early treatment and prevention of cancer [44].

Health promotion intervention is based on our beliefs, understandings and theories of the determinants of behaviors. As proposed by McLeroy in the ecological model, behaviors are determined and influenced by five factors: Intrapersonal, interpersonal, institutional, community and public policy [45]. This study also suggests that multiple strategies might be needed to change an individual’s health behavior. Apart from an individual’s attitudes and self-concept, support systems, social rules and regulations, peer groups, informal networks, national laws and policies can be employed to understand the causes and possible actions to modify health-related behavior changes.

This study also has some limitations. First, since this is a cross-sectional study, it cannot be used to analyze behavior over a longer period to time. Second, convenience sampling was used for data collection in this study, which might have caused biases.

## 5. Conclusions

Our study demonstrated a lack of awareness about cervical cancer and HPV infection among Nepalese adolescents and their mothers. There was no association between knowledge of students and screening practices of mothers; however, cancer communication between mothers and students helped to increase awareness regarding cervical cancer. Talk on cancer was strong predictor of knowledge among students. Hence, students and mothers’ communication should be increased to reduce the knowledge gap between parents and children. There is a strong need for a government- supported, school-based cancer education program to address the issues of HPV vaccination, cervical cancer screening and the reduction of risk factors. Most of the participants were unaware of the HPV vaccine. Mothers who have had cervical cancer screening are not able to maintain the screening practice. Unless a trigger is present, mothers are less likely to take and continue screening tests. Programs conducted involving adolescents, peer groups and their family members are more effective and acceptable when both groups are comfortable in sharing their ideas or opinions. In order to check the association of knowledge of students and screening practice of mothers, fathers were not included in this study. Since fathers and husbands play an influential role in female’s decision to screening, further studies are important to understand the knowledge as well as attitudes of males on cervical cancer and screening behaviors. An education program, by collaborating with family members and students, might help to address the issue of the HPV vaccine for students and screening tests for mothers.

## Figures and Tables

**Table 1:** Relationship between the demographic variables of students and the total cervical cancer knowledge score.

Variables	Category (*N*)	Mean ± Standard Deviation	*p*-Value
Sex	Male (125)	10.4 ± 5.2	0.01
	Female (128)	11.9 ± 3.9	
Education Level	Grade 9 (137)	10.5 ± 4.7	0.02
	Grade 10 (116)	11.8 ± 4.3	
Area of Residence	Rural (79)	10.4 ± 5.0	0.10
	Urban (174)	11.4 ± 4.5	
Healthy Diet Consumption Per Week	Not much (30)	8.9 ± 5.5	0.00
	Sometimes (76)	11.0 ± 4.9	
	3 to 4 times (106)	11.9 ± 3.8	
	Every day (41)	11.9 ± 4.9	
Family History of Illness	No (197)	11.1 ± 4.6	0.88
	Yes (56)	11.2 ± 4.8	
Cancer Talk with Others	No (187)	10.6 ± 4.9	0.00
	Yes (66)	12.6 ± 3.4	
Mother’s Age during Marriage	Before 20 years (164)	10.9 ± 4.6	0.31
	After 20 years (89)	11.6 ± 4.6	
Mother’s Cancer Screening Practice	No (215)	10.7 ± 5.2	0.56
	Yes (38)	11.4 ± 4.6	

*N* = 253. *t*-test, ANOVA; Total knowledge score range: 0–21.

**Table 2:** Multiple linear regression to identify the predictors of awareness on students.

Variables	B	*β*	*p*-Value
Sex ^a^	1.45	0.16	0.01
Cancer Talk with Others ^b^	2.01	0.19	0.00
Education Level ^c^	1.49	0.19	0.01
Healthy Diet Consumption ^d^	0.65	0.13	0.04

^a^ [1: Female], ^b^ [1: Yes], ^c^ [1: Grade 10], ^d^ [4: Every day]. Dependent variable: Total cervical cancer knowledge score.

**Table 3:** Relationship between the demographic variables of mothers and the total cervical cancer knowledge score.

Variables	Category (*N*)	Mean ± SD	*p*-Value
Age	Less than 49 years (225)	10.2 ± 4.9	0.43
	Above 49 years (28)	11.0 ± 3.8	
Education Level	Illiterate (104)	9.9 ± 5.5	0.00
	Up to grade 8 (39)	8.9 ± 4.4	
	Up to grade 12 (86)	11.5 ± 3.7	
	University and above (24)	13.2 ± 3.1	
Number of Children	Less than 3 (177)	10.5 ± 4.7	0.51
	More than 3 (76)	10.0 ± 4.9	
Area of Residence	Rural (79)	9.0 ± 5.2	0.00
	Urban (174)	10.9 ± 4.5	
Smoking Habit	No (226)	10.3 ± 4.8	0.96
	Yes (27)	10.3 ± 4.9	
Drinking Habit	No (194)	10.3 ± 5.0	0.89
	Yes (59)	10.4 ± 3.9	
Family History of Illness	No (178)	10.4 ± 4.6	0.84
	Yes (51)	10.5 ± 5.1	
Exercise Habit Per Week	No (200)	9.8 ± 4.9	0.00
	Sometimes (18)	11.6 ± 3.5	
	3 to 4 times (9)	11.9 ± 3.4	
	Every day (26)	13.2 ± 3.3	
Healthy Diet Consumption	No (24)	8.9 ± 4.1	0.08
	Yes (229)	10.5 ± 4.8	
Cancer Screening Habit	No (215)	9.9 ± 4.9	0.01
	Yes (38)	12.8 ± 3.0	
Talk on Cancer	No (152)	9.8 ± 5.0	0.02
	Yes (101)	11.2 ± 4.2	
Age during Marriage	Before 20 years (164)	9.8 ± 5.0	0.01
	After 20 years (89)	11.4 ± 4.2	
Sex of Children	Male (125)	9.3 ± 4.2	0.05
	Female (128)	10.9 ± 4.5	

*N* = 253. *t*-test, ANOVA; Total knowledge score range: 0–21.

**Table 4:** Multiple linear regression to identify the predictors of awareness on mothers.

Variables	B	*β*	*p*-Value
Area of Residence ^a^	1.29	0.13	0.04
Exercise Habit ^b^	0.80	0.16	0.01
Talk on Cancer ^c^	0.33	0.03	0.58
Age during Marriage ^d^	0.70	0.07	0.26
Sex of Children ^e^	1.07	0.11	0.06
Cancer Screening Habit ^f^	2.23	0.17	0.01
Education Level ^g^	0.66	0.15	0.03

^a^ [1: Urban], ^b^ [4: Every day], ^c^ [1: Yes, talked], ^d^ [1: More than 20 years], ^e^ [1: Female], ^f^ [1: Yes], ^g^ [4: University and above]. Dependent Variable: Total Cervical Cancer Knowledge Score.

**Table 5:** Relationship between the total cervical cancer knowledge score and the barriers to screening perceived by mothers.

SN	Barriers	Category (*N*)	Mean ± SD	*p*-Value
1	I have low risk of cervical cancer.	No (67)	12.1 ± 4.1	0.00
		Yes (186)	9.7 ± 4.8	
2	I will get stressed after finding the result of cancer screening.	No (242)	12.7 ± 4.5	0.08
		Yes (11)	10.2 ± 4.7	
3	Screening is not necessary since there is no certainty of cancer.	No (238)	11.1 ± 4.9	0.54
		Yes (15)	10.3 ± 4.7	
4	Screening test is expensive.	No (247)	11.7 ± 4.0	0.28
		Yes (6)	10.3 ± 4.7	
5	I don’t know where to go for screening.	No (214)	10.5 ± 4.7	0.09
		Yes (39)	9.1 ± 5.1	
6	I don’t have time to go for screening.	No (240)	10.5 ± 4.8	0.04
		Yes (13)	7.7 ± 5.3	
7	I don’t have much information about screening.	No (122)	11.6 ± 4.2	0.00
		Yes (131)	9.2 ± 4.9	

*N* = 253. *t*-test; Total knowledge score range: 0–21.

## References

[1] WHO (2018). Cervical Cancer, Cancer. http://www.who.int/cancer/prevention/diagnosis-screening/cervical-cancer/en/.

[2] WHO (2018). Cancer Country Profile: Nepal. http://www.who.int/cancer/country-profiles/npl_en.pdf.

[3] Nepal Network for Cancer Treatment & Research (NNCTR) (2010). Cervical and Breast Cancer Screening Activities in Nepal. https://www.globalgiving.org/pfil/6686/projdoc.pdf.

[4] Bruni L., Albero G., Serrano B., Mena M., Gomez D., Munoz J., Bosch F.X., de Sanjose S. (2019). Papillomavirus and Related Diseases in Nepal.

[5] Gyawali B., Keeling J., Teijlingen E., Dhakal L., Aro A.R. (2015). Cervical cancer screening in Nepal: Ethical considerations. Med. Bioeth..

[6] Johnson D.C., Bhatta M.P., Smith J.S., Kempf M.C., Boker T.R., Vermund S.H., Chamot E., Aryal S., Lhaki P., Shrestha S. (2014). Assessment of high-risk human papillomavirus infections using clinician- and self-collected cervical sampling methods in rural women from far western Nepal. PLoS ONE.

[7] Joshi M., Mishra S.R. (2013). Cervical cancer screening in Nepal. J. Public Health.

[8] WHO (2019). Screening for Cervical Cancer, Cancer. https://www.who.int/cancer/detection/cervical_cancer_screening/en/.

[9] Seng L.M., Rosman A.N., Khan A., Haris N.M., Mustapha N.A.S., Husaini N.S.M., Zahari N.F. (2018). Awareness of cervical cancer among women in Malaysia. Int. J. Health Sci..

[10] American Cancer Society (2019). What are the Risk Factors for Cervical Cancer?. https://www.cancer.org/cancer/cervical-cancer/causes-risks-prevention/risk-factors.html.

[11] Darj E., Chalise P., Shakya S. (2019). Barriers and facilitators to cervical cancer screening in Nepal: A qualitative study. Sex. Repro Healthc..

[12] Yamaguchi N., Tsukamoto Y., Shimoyama H., Nakayama K., Misawa S. (2011). Effects of peer education interventions aimed at changing awareness of cervical cancer in nursing students. Niigata J. Health Welf..

[13] UNICEF (2018). Ending child marriage in Nepal. https://www.unicef.org/rosa/media/151/file.

[14] Kyle R.G., Nicoll A., Forbat L., Hubbard G. (2013). Adolescents’ awareness of cancer risk factors and associations with health-related behaviors. Health Educ. Res..

[15] Central Bureau of Statistics, Government of Nepal (2011). National Population and Housing Census (National Report). https://unstats.un.org/unsd/demographic-social/census/documents/Nepal/Nepal-Census-2011-Vol1.pdf.

[16] Rashid S., Labani S., Das B.C. (2016). Knowledge, awareness and attitude on HPV, HPV vaccine and cervical cancer among college students in India. PLoS ONE.

[17] Takata T.E., Ueda Y., Morimoto A., Yoshino K., Kimura T., Nishikawa N., Sekine M., Horikoshi Y., Takagi T., Enomoto T. (2015). Survey of Japanese mothers of daughters eligible for human papillomavirus vaccination on attitudes about media and reports of adverse events and the suspension of governmental recommendation for vaccination. J. Obstet. Gynaecol. Res..

[18] Sawada M., Ueda Y., Yagi A., Morimoto A., Nakae R., Kakubari R., Abe H., Takata T.E., Iwamiya T., Matsuzaki S. (2018). HPV vaccination in Japan: Results of a 3 year follow up survey of obstetricians and gynecologists regarding their opinions toward the vaccine. Int. J. Clin. Oncol..

[19] Chang S.C.H., Woo J.S.T., Gorzalka B.B., Brotto L.A. (2010). A questionnaire study of cervical cancer screening beliefs and practices of chinese and caucasian mother- daughter pairs living in Canada. J. Obstet. Gynaecol. Can..

[20] Turner L.W., Hunt S.B., Dibrezzo R.O., Jones C. (2004). Design and implementation of an osteoporosis prevention program using the health belief model. Am. J. Health Stud..

[21] Janz N.K., Becker M.H. (1984). The Health Belief Model: A decade later. Health Educ. Q..

[22] Jaglarz K., Tomaszewski K.A., Kamzol W., Puskulluoglu M., Krzemieniecki K. (2014). Creating and field testing the questionnaire for the assessment of knowledge about cervical cancer and its prevention among schoolgirls and female students. J. Gynaecol Oncol..

[23] Mulatu K., Motma A., Seid M., Tadesse M. (2017). Assessment of knowledge, attitude and practice on cervical cancer screening among female students of Mizan Tepi University. Cancer Biol. Ther. Oncol..

[24] Adhikari R., Tamang J. (2009). Premarital sexual behavior among male college students of Kathmandu, Nepal. BMC Public Health.

[25] Sugisaki K., Ueda S., Monobe H., Suketomo H.Y., Eto T., Waanabe M., Mori R. (2014). Cancer understanding among Japanese students based on a national wide survey. Environ. Health Prev. Med..

[26] Singh Y., Vaidya P. (2010). Human Papilloma virus vaccination in Nepal. Asian Pac. J. Cancer Prev..

[27] Mouallif M., Bowyer H., Festali S., Albert A., Filali Y., Guenin S., Delvenne P., Waller J., Ennaji M. (2014). Primary cervical cancer prevention in Morocco: HPV vaccine awareness and acceptability among parents. Procedia Vaccinol..

[28] Ministry of Finance, Government of Nepal Budget Speech of Fiscal Year 2019/2020. https://mof.gov.np/uploads/document/file/budget_speech_website_20190619052055.pdf.

[29] Hardcastle S.J., Hancox J., Hattar A., Maxwell- Smith C., Ntoumani C.T., Hagger M.S. (2015). Motivating the unmotivated: How can health behavior be changed in those unwilling to change?. Front. Psychol..

[30] Pitts M.K., Dyson S.J., Rosenthal D.A., Garland S.M. (2007). Knowledge and awareness of human papillomavirus: Attitudes towards HPV vaccination among a representative sample of women in Victoria, Australia. Sex. Health.

[31] Rosser J., Zakaras J.M., Hamisi S., Huchko M.J. (2014). Men’s knowledge and attitudes about cervical cancer screening in Kenya. BMC Women’s Health.

[32] Liu T., Li S., Ratcliffe J., Chen G. (2017). Assessing knowledge and attitudes towards cervical cancer screening among rural women in eastern china. Int. J. Environ. Res. Public Health.

[33] Pandey R.A., Karmacharya E. (2017). Cervical cancer screening behavior and associated factors among women of Ugrachandi Nala, Kavre, Nepal. Eur. J. Med. Res..

[34] Poudel K., Sumi N. (2017). Health behavior regarding Cardiovascular Diseases among Nepali adults. J. Community Health.

[35] Shrestha S., Saha R., Tripathi N. (2013). Knowledge, attitude and practice regarding cervical cancer screening amongst women visiting tertiary center in Kathmandu, Nepal. Nep. J. Med. Sci..

[36] Ranjit A., Gupta S., Shrestha R., Kushner A.L., Nwomeh B.C., Groen R.S. (2016). Awareness and prevalence of cervical cancer screening among women in Nepal. Int. J. Gynecol. Obstet..

[37] WHO (2012). Prevention of Cervical Cancer through Screening Visual Inspection with Acetic Acid (VIA) and Treatment with Cryotherapy. http://www.who.int/reproductivehealth/publications/cancers/9789241503860/en/.

[38] WHO (2013). Comprehensive Cervical Cancer Prevention and Control: A Healthier Future for Girls and women. https://apps.who.int/iris/bitstream/handle/10665/78128/9789241505147_eng.pdf;jsessionid=79B630E91CD70A0714AB076D4CEB7DB6?sequence=3.

[39] Thapa N., Maharjan M., Petrini M.A., Shah R., Shah S., Maharjan N., Shrestha N., Cai H. (2018). Knowledge, attitude, practice and barriers of cervical cancer screening among women living in mid- western rural, Nepal. J. Gynecol. Oncol..

[40] Navarro A.M., Raman R., McNicholas L.J., Loza O. (2007). Diffusion of cancer education information through a Latino Community Health Advisor Program. Prev. Med..

[41] Poudel K., Sumi N. (2018). Knowledge about risk factors for cancer among adults in Nepal. KnE Life Sci..

[42] Ncube B., Bey A., Knight J., Bessler P., Jolly P.E. (2015). Factors associated with the uptake of cervical cancer screening among women in Portland, Jamaica. N. Am. J. Med. Sci..

[43] Mishra S.R., Khanal P., Karki D.K., Kallestrup P., Enemark U. (2015). National health insurance policy in Nepal: Challenges for implementation. Glob. Health Action.

[44] Tapera R., Manyala E., Erick P., Maswabi T.M., Tumoyagae T., Mbongwe B., Letsholo B. (2017). Knowledge and Attitudes towards Cervical cancer screening amongst University of Botswana female students. Asian Pac. J. Cancer Prev..

[45] McLeroy K.R., Bibeau D., Steckler A., Glanz K. (1988). An ecological perspective on health promotion programs. Health Educ. Q..

